# Associations between Plasma Metabolites, Dementia and Modifiable Risk Factors

**DOI:** 10.1002/alz70856_106389

**Published:** 2026-01-10

**Authors:** Yue Liu, Asger Wretlind, Dorsa Abdolkarimi, Sheena Waters, Charles R Marshall, Cristina Legido‐Quigley, Petroula Proitsi

**Affiliations:** ^1^ Queen Mary University of London, London, Greater London, United Kingdom; ^2^ Institute of Psychiatry, Psychology and Neuroscience, Maurice Wohl Clinical Neuroscience Institute, King's College London, London, United Kingdom; ^3^ Wolfson Institute of Populational Heath, Queen Mary University of London, London, Greater London, United Kingdom; ^4^ Centre for Preventive Neurology, Wolfson Institute of Population Health, Queen Mary University of London, London, United Kingdom

## Abstract

**Background:**

Metabolic dysfunction is increasingly recognized as an early contributor to the pathophysiology of dementia. Identifying blood‐based biomarkers associated with preclinical disease changes may provide valuable insights into early disease mechanisms and potential intervention strategies. This study aimed to determine sparse metabolite signatures of dementia risk and to determine whether metabolites mediate the relationship between established modifiable risk factors and dementia using both observational and genetic epidemiology approaches.

**Method:**

This study included 271,610 participants from the UK Biobank (UKB), with 327 plasma metabolomic measures (168 absolute concentrations and 159 derived ratios) quantified per sample. A lasso‐regularized Cox proportional hazards regression model was used to identify a signature of metabolites associated with the risk of all‐cause dementia (ACD), vascular dementia (VaD), and Alzheimer's disease (AD). The metabolomic risk score (MRS) derived from metabolite signatures was evaluated for its association with brain structural changes. Mediation analysis was performed to assess whether metabolite signatures mediated the associations between established dementia risk factors and incident dementia. Finally, Mendelian randomization (MR) analyses including Multivariable MR were conducted to explore potential causal associations and mediating effect.

**Result:**

Metabolomic signatures predicted ACD, AD, and VaD with C‐indices of 0.63, 0.65, and 0.69, respectively, and significantly improved predictive performance when adding to clinical factors (C‐indices improvement 0.002‐0.003). The VaD and ACD MRS showed widespread associations with most regional brain volumes and white matter microstructures. Glucose and the percentage of linoleic acid (LA%) were primarily responsible for mediating the association between obesity and ACD/VaD, with the proportion of mediation exceeding 30%. In MR analysis, albumin and glutamine were nominally associated with AD, and LA% was nominally associated with VaD. Finally, LA% significantly mediated the causal relationship between diabetes and VaD.

**Conclusion:**

Metabolomic signatures improved dementia prediction beyond established clinical risk factors and mediated the relationship between these risk factors and dementia. MRS were strongly associated with volume changes in multiple brain regions. This study implicates early metabolic pathobiology in the development of dementia and may inform potential intervention strategies. Future research will employ advanced MR methods to further characterise these complex relationships.

